# Multifactorial Hypercalcemia and Literature Review on Primary Hyperparathyroidism Associated with Lymphoma

**DOI:** 10.1155/2014/893134

**Published:** 2014-03-05

**Authors:** Jelena Maletkovic, Jennifer P. Isorena, Miguel Fernando Palma Diaz, Stanley G. Korenman, Michael W. Yeh

**Affiliations:** ^1^Department of Endocrinology, UCLA David Geffen School of Medicine, Los Angeles, CA 90095, USA; ^2^Section of Endocrine Surgery, David Geffen School of Medicine at UCLA, Los Angeles, CA 90095, USA; ^3^Department of Pathology and Laboratory Medicine, UCLA David Geffen School of Medicine, Los Angeles, CA 90095, USA

## Abstract

The most common cause of hypercalcemia in hospitalized patients is malignancy. Primary hyperparathyroidism most commonly causes hypercalcemia in the outpatient setting. These two account for over 90% of all cases of hypercalcemia. Hypercalcemia can be divided into PTH-mediated and PTH-independent variants. Primary hyperparathyroidism, familial hypocalciuric hypercalcemia, familial hyperparathyroidism, and secondary hyperparathyroidism are PTH mediated. The most common PTH-independent type of hypercalcemia is malignancy related. Several mechanisms lead to hypercalcemia in malignancy-direct osteolysis by metastatic disease or, more commonly, production of humoral factors by the primary tumor also known as humoral hypercalcemia of malignancy that accounts for about 80% of malignancy-related hypercalcemia. The majority of HHM is caused by tumor-produced parathyroid hormone-related protein and less frequently production of 1,25-dihydroxyvitamin D or parathyroid hormone by the tumor. We report the rare case of a patient with hypercalcemia and diagnosed primary hyperparathyroidism. The patient had persistent hypercalcemia after surgical removal of parathyroid adenoma with recorded significant decrease in PTH level. After continued investigation it was found that the patient also had elevated 1,25-dihydroxyvitamin D and further studies confirmed a large spleen mass that was later confirmed to be a lymphoma. This is a rare example of two concomitant causes of hypercalcemia requiring therapy.

## 1. Introduction

Hypercalcemia is defined as total serum calcium above 10.5 mg/dL (>2.6 mmol/L). In a pregnant patient the upper limit of normal is considered to be 9.5 mg/dL [1]. The most common cause of hypercalcemia in hospitalized patients is malignancy. Primary hyperparathyroidism most commonly causes hypercalcemia in the outpatient setting [2]. These two account for over 90% of all cases of hypercalcemia.

Hypercalcemia can be divided into PTH-mediated and PTH-independent variants. Primary hyperparathyroidism (PHPT), familial hypocalciuric hypercalcemia, familial hyperparathyroidism, and secondary hyperparathyroidism are PTH mediated. In these patients initial evaluation reveals increased or inappropriately normal PTH (not suppressed in the setting of hypercalcemia) which narrows the differential diagnosis. The most common PTH-independent type of hypercalcemia is malignancy related. Several mechanisms lead to hypercalcemia in malignancy-direct osteolysis by metastatic disease or, more commonly, production of humoral factors by the primary tumor also known as humoral hypercalcemia of malignancy (HHM) that accounts for about 80% of malignancy-related hypercalcemia. The majority of HHM is caused by tumor-produced parathyroid hormone-related protein (PTHrP) and, less frequently, production of 1,25-dihydroxyvitamin D (1,25D) or parathyroid hormone by the tumor [3]. Tumors secreting PTHrP cause increased bone resorption and distal renal tubular calcium reabsorption. Tumors that cause elevation in 1,25D cause hypercalcemia as a result of a combination of increased bone resorption and intestinal calcium absorption.

Primary hyperparathyroidism with an adenoma or hyperplasia producing hypercalcemia is a relatively common endocrine problem that is treated surgically. If the hypercalcemia persists after resection of an adenoma, then the differential diagnosis would depend on whether the hypercalcemia is associated with a relatively high or a low PTH. With a high PTH the surgeon needs to seek another adenoma or parathyroid hyperplasia. With a low PTH, hypercalcemia must depend on other causes that have to be sought.

We report the rare case of a patient with two concomitant causes of hypercalcemia requiring therapy.

## 2. Case

A previously healthy 67-year-old man was taken to the emergency room for polyuria, unsteady gait, dizziness, and confusion. The patient was found to have a calcium level of 16.3 mg/dL (reference range 8.6–10.2) and acute renal failure with a Cr of 4.9 mg/dL. The PTH level was elevated at 58 pg/mL (11–51 pg/mL). He was treated with IV fluids, calcitonin, and one dose of pamidronate. His serum calcium and creatinine improved. An ultrasound of the neck was consistent with a right inferior parathyroid mass that on surgical removal was confirmed by histopathology to be a parathyroid adenoma. Intraoperatively his PTH fell from 51 to 10 pg/mL. The serum calcium at 11.1 mg/dL did not normalize following the surgery.

His creatinine became normal and the PTH remained low at 5 pg/mL. Workup for persistent hypercalcemia revealed a normal PTHrP and a high 1,25-dihydroxyvitamin D of >200 pg/mL (reference range: 15–75) indicating the additional mechanism of hypercalcemia (Table 1).

**Figure 1:**
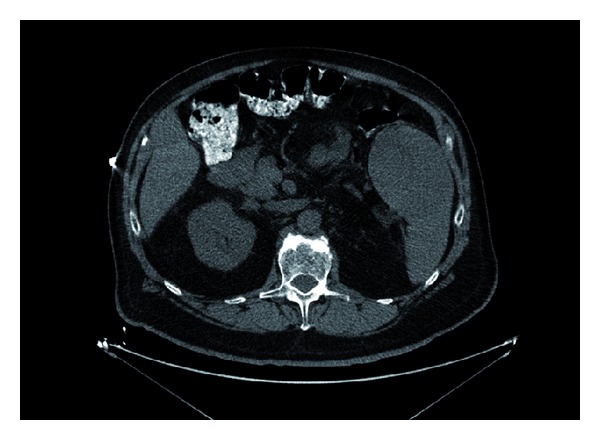
Abdominal CT showing splenic mass.

His previous workup was negative for sarcoidosis and multiple myeloma and he never took any vitamin D or calcium supplements. An abdominal CT scan showed a splenic mass (Figure 1).

**Figure 2:**
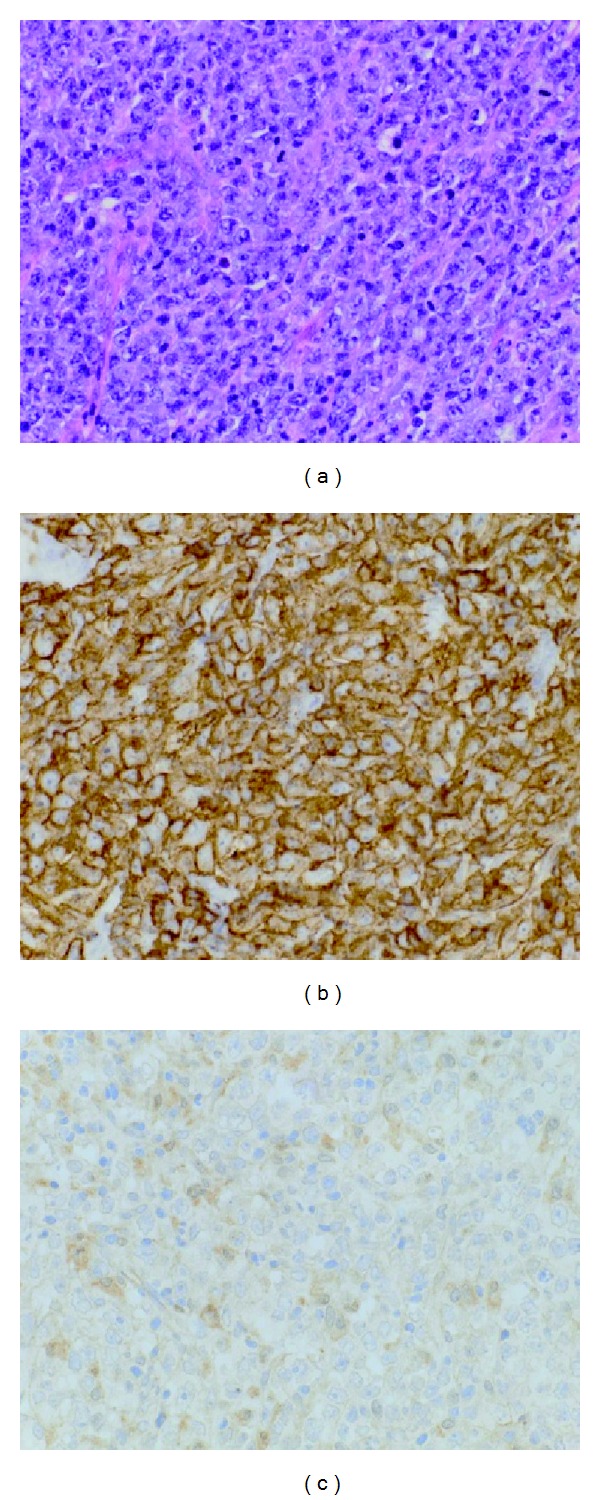
(a) H&E, 40x: sections of the spleen show a proliferation of highly atypical large lymphoid cells resulting in extensive effacement of the splenic architecture. The cells show irregular nuclei with open vesicular chromatin, prominent nucleoli, and moderate to scant cytoplasm. Abundant mitotic figures are seen. (b) Immunohistochemistry, CD20, 40x: the neoplastic lymphoid cells exhibit diffuse and strong staining for CD20. (c) Immunohistochemistry, 1-alpha-hydroxylase, 40x: neoplastic lymphoid cells exhibit moderate, irregular immunoreactivity for 1-alpha-hydroxylase in a cytoplasmic distribution.

Following the resection of this mass the calcium level normalized. The mass was found to be a diffuse large B-cell lymphoma. Immunohistochemistry showed that neoplastic lymphoid cells exhibit strong staining for CD20. Most importantly, from the hypercalcemia perspective, it shows immunoreactivity for 1-alpha-hydroxylase in a cytoplasmic distribution (Figures 2(a), 2(b), and 2(c)).

## 3. Discussion

Hypercalcemia is a relatively frequent complication of lymphoma [4]. Increased circulating levels of 1,25D have been described in patients with Hodgkin's and non-Hodgkin's lymphomas [5, 6]. Calcidiol (25-hydroxyvitamin D) is metabolically activated in the proximal convoluted tubule cells of the kidney to produce calcitriol (1,25D) by 1-alpha hydroxylase. This reaction is stimulated by PTH, calcitonin, and hypophosphatemia and inhibited by calcium, 1,25D, and hyperphosphatemia [7]. 1-Alpha-hydroxylase belongs to the cytochrome p450 superfamily of enzymes and is encoded by the CYP27B1 gene.

Hewison and colleagues used immunohistochemistry to demonstrate the presence of 1*α*-hydroxylase in macrophages associated with lymphomas. They suggested that the tumor secreted factors that stimulated 1*α*-hydroxylase expression by macrophages [8].

Some studies have claimed that 15% of patients with hypercalcemia and malignancy have coexisting hyperparathyroidism (Table 2). A report by Strodel et al. in 1988 shows a group of 18 patients with malignant tumors and hypercalcemia caused by PHPT. In all cases, serum levels of calcium returned to normal after surgical removal of a parathyroid adenoma or hyperplasia indicating that the hypercalcemia was caused by only one mechanism [9]. Hutchesson et al. report 47 patients with hypercalcemia and malignancy; however, in three patients it was found that hypercalcemia was caused by PHPT and hypercalcemia resolved after surgical removal of a parathyroid adenoma [10]. Owen et al. report 2 cases of parathyroid adenoma in patients with primary cutaneous lymphomas [11]. In both cases serum calcium levels returned to normal after removal of the parathyroid adenoma. Neither PTHrP nor 1,25D levels were assessed. Two additional isolated cases of parathyroid adenoma causing hypercalcemia in patients with T-cell lymphoma [12] and lymphomatoid papulosis [13] were reported subsequently.

In all but two reports the hypercalcemia was caused by only one mechanism. A patient of Gallacher et al. was found to have persistent hypercalcemia after a parathyroid adenoma was removed and PTH levels fell, leading to further investigation that revealed elevated PTHrP and a carcinoma in the manubrium [14]. The humoral mechanism via PTHrP, however, was different than our patient's who had 1,25D elevation. The only report to our knowledge of coexisting PHPT and diffuse large B-cell lymphoma causing 1,25D elevation and multifactorial hypercalcemia was published in 2013 by Luceri and Haenel IV [15].

Two distinct causes of hypercalcemia may coexist by chance. Some authors have also suggested that hyperparathyroidism may be associated with the slight increase in the incidence of malignancy [16]. At this time we do not know whether there is any relation between lymphoma and PHPT.

Persistence of hypercalcemia after removal of the causal lesion should prompt a thorough workup for another source based on whether the offending agent is PTH, PTHrP, or Vitamin D.

## Figures and Tables

**Table 1:** Pertinent labs before and after parathyroidectomy and resection of the spleen.

Lab	On first presentation	After parathyroidectomy Postop. day 1	After parathyroidectomy Postop. day 10	After splenectomy	~One year after splenectomy
Total calcium (mg/dL)	16.3	11.1	12.9	10.6	8.9
PTH (pg/mL)	58	5	4	5	78
Creatinine (mg/dL)	4.9	0.9	1.8	1.3	1.29
25D (pg/mL)	22	19	19	18.5	37
1.25D (pg/mL)			>220	133.6	62
PTHrP (pmol/L)			<2		

**Table 2:** The literature review of reported cases of hypercalcemia and malignancy.

Study	Basic study features	Cause of hypercalcemia
Strodel et al. [9]	18 patients with malignancy and hypercalcemia	All patients had PHPT as the only cause of hypercalcemia.
Hutchesson et al. [10]	47 patients with malignancy and hypercalcemia	3 of 47 patients had PHPT as the cause of hypercalcemia. No cases of 2 causes of elevated calcium levels.
Owen et al. [11]	2 cases of primary cutaneous lymphomas and hypercalcemia	Both cases were found to have PHPT as the only cause of hypercalcemia
Albès et al. [12]	A case of T-cell lymphoma and hypercalcemia	Hypercalcemia caused by PHPT only
Aguilar-Bernier et al. [13]	Lymphomatoid papulosis	Hypercalcemia caused by PHPT only
Gallacher et al. [14]	Hypercalcemia did not resolve after removal of parathyroid adenoma which prompted further workup and the patient was found to have malignancy in the manubrium sterni	One case with two coexisting mechanisms of hypercalcemia—PHPT and PTHrP mediated
Luceri and Haenel [15]	Hypercalcemia did not resolve after removal of parathyroid adenoma which prompted further workup and the patient was found to have diffuse large B-cell lymphoma	One case with two coexisting mechanisms of hypercalcemia—PHPT and 1,25-dihydroxyvitamin D mediated
